# Feasibility of optical coherence tomography angiography to assess changes in retinal microcirculation in ovine haemorrhagic shock

**DOI:** 10.1186/s13054-018-2056-3

**Published:** 2018-05-29

**Authors:** Maged Alnawaiseh, Christian Ertmer, Laura Seidel, Philip Helge Arnemann, Larissa Lahme, Tim-Gerald Kampmeier, Sebastian Willy Rehberg, Peter Heiduschka, Nicole Eter, Michael Hessler

**Affiliations:** 1 Department of Ophthalmology, University of Muenster Medical Centre, Domagkstraße 15, 48149 Muenster, Germany; 2 Department of Anaesthesiology, Intensive Care, and Pain Therapy, University of Muenster Medical Centre, Albert-Schweitzer-Campus 1, Building A1, 48149 Muenster, Germany; 3 Department of Anaesthesiology, University of Greifswald, Ferdinand-Sauerbruch-Straße, 17475 Greifswald, Germany

**Keywords:** Fluid therapy, Haemorrhagic shock, Microcirculation, Optical coherence tomography angiography, Sheep

## Abstract

**Background:**

This study aimed to investigate the feasibility of optical coherence tomography angiography (OCT-A) for quantitative analysis of flow density to assess changes in retinal perfusion in an experimental model of haemorrhagic shock.

**Methods:**

Haemorrhagic shock was induced in five healthy, anaesthetized sheep by stepwise blood withdrawal of 3 × 10 ml∙kg^− 1^ body weight. OCT-A imaging of retinal perfusion was performed using an OCT device. Incident dark-field illumination microscopy videos were obtained for the evaluation of conjunctival microcirculation. Haemodynamic variables and flow density data in the OCT angiogram were analysed before and during progressive haemorrhage resulting in haemorrhagic shock as well as after fluid resuscitation with 10 ml∙kg^− 1^ body weight of balanced hydroxyethyl starch solution (6% HES 130/0.4). Videos of the conjunctival microcirculation were recorded at baseline, in haemorrhagic shock, and after resuscitation. Data are presented as median with interquartile range. Comparisons between time points were made using Friedman’s test and the degree of correlation between two variables was expressed as Spearman’s rank correlation coefficient.

**Results:**

Mean arterial pressure and cardiac index (CI) decreased and lactate concentration increased after induction of shock, and haemodynamics recovered after resuscitation. The flow density in the superficial retinal OCT angiogram decreased significantly after shock induction (baseline 44.7% (40.3; 50.5) vs haemorrhagic shock 34.5% (32.8; 40.4); *P* = 0.027) and recovered after fluid resuscitation (46.9% (41.7; 50.7) vs haemorrhagic shock; *P* = 0.027). The proportion of perfused vessels of the conjunctival microcirculation showed similar changes. The flow density measured using OCT-A correlated with the conjunctival microcirculation (perfused vessel density: Spearman’s rank correlation coefficient ρ = 0.750, *P* = 0.001) and haemodynamic parameters (CI: ρ = 0.693, *P* < 0.001).

**Conclusions:**

Retinal flow density, measured using OCT-A, significantly decreased in shock and recovered after fluid therapy in an experimental model of haemorrhagic shock. OCT-A is feasible to assess changes in retinal perfusion in haemorrhagic shock and fluid resuscitation.

**Electronic supplementary material:**

The online version of this article (10.1186/s13054-018-2056-3) contains supplementary material, which is available to authorized users.

## Background

Blood flow in the microcirculation (vessels smaller than 100 μm) plays an important role in the delivery of oxygen and nutrients to cells. Maintaining adequate blood flow to the microcirculation is a prerequisite for normal organ perfusion and function. In critically ill patients, capillary blood flow is often impaired and changes in capillary perfusion are associated with outcome [1–4].

In acute haemorrhagic shock, excessive blood loss results in depressed cardiac output, which leads to decreased tissue perfusion with tissue hypoxia, organ dysfunction, and finally death. However, routine clinical parameters, which often serve as surrogates for monitoring of tissue perfusion in daily clinical practice (e.g. cardiac output, arterial blood lactate, heart rate, or urine output), do not always adequately reflect the level of tissue perfusion, and optimizing these parameters does not necessarily ensure adequate tissue perfusion [5–9]. In consequence, there is a need to introduce direct parameters of tissue perfusion (i.e. microcirculatory parameters) to support therapy decisions and to guide therapy in the future [5].

Using various novel techniques, it is possible to monitor patients’ microcirculation directly at the bedside. Research using these new techniques has demonstrated the important role of the microcirculation in critical diseases [3, 4]. The microcirculation in critically ill patients has been evaluated in the splanchnic region, skin, muscles, and the sublingual area [3]. In contrast, to our knowledge only a few studies evaluated changes in microvascular perfusion of the retina in critically ill patients, for example in sepsis [10].

The retina is an embryological projection of the forebrain. Impaired retinal perfusion might indicate reduced cerebral blood flow, since the ophthalmic artery arises from the internal carotid artery. Previous studies have shown a close relationship between cerebral events and qualitative measurements of retinal microvascular abnormalities [11–15].

Optical coherence tomography angiography (OCT-A) is a relatively novel technology, which provides high-resolution images of the retinal and choroidal vascularization. This method is non-invasive, reproducible, and can be quickly performed at the bedside. In consequence, this technology has attracted increasing interest over the last 2 years. It has been described in healthy subjects, in a range of ocular and systemic diseases in humans, and in various animal models [16–24].

The purpose of the current study is to evaluate the feasibility of OCT-A to assess the changes in retinal microcirculation in shock and fluid resuscitation in an experimental model of haemorrhagic shock in sheep.

## Methods

### Anaesthesia and instrumentation

After approval by the local veterinary authority (North Rhine-Westphalia State Environment Agency), five healthy female sheep (*Ovis orientalis aries*) with median (interquartile range) body weight of 53.7 (48.8; 55.1) kg were anaesthetized by intramuscular bolus injection of S-ketamine (10 mg∙kg^− 1^) and midazolam (0.3 mg·kg^− 1^). The sheep were intubated endotracheally and mechanically ventilated throughout the experiment with a tidal volume of 10 ml∙kg^− 1^ body weight, targeting an end tidal carbon dioxide pressure of 35 ± 5 mmHg by adjusting the respiratory rate. General anaesthesia and analgesia were maintained by continuous infusion of midazolam (0.3 mg·kg^− 1^·h^− 1^) and S-ketamine (1 mg·kg^− 1^·h^− 1^) throughout the experiment.

For haemodynamic monitoring and blood withdrawal, sheep were instrumented with a pulse contour cardiac output catheter (5-Fr PiCCO™ catheter; Pulsion Medical Systems, Munich, Germany) in the left femoral artery, a central venous line in the right jugular vein, and a 7.5-French catheter in the left jugular vein. A Foley catheter in the urinary bladder was used to measure urine output.

### Experimental protocol

Haemorrhagic shock was induced in the five sheep by withdrawal of 3 × 10 ml blood per kilogram of body weight according to an established protocol [25, 26], which corresponds to approximately 50–60% of total blood volume in sheep [27]. After each step of blood withdrawal, a recovery period of 30 min was introduced to allow homeostasis to occur at the current level of hypovolemia. If the mean arterial pressure consistently dropped below 30 mmHg, the current withdrawal step was stopped for safety reasons (i.e. to prevent death) and the recovery period was started. After the third stage of blood withdrawal, haemorrhagic shock measurements were performed. After haemorrhagic shock measurements, fluid resuscitation was performed with an intravenous infusion of 10 ml∙kg^− 1^ bodyweight of balanced hydroxyethyl starch solution (6% HES 130/0.4) within 30 min. Another set of measurements was taken after fluid resuscitation.

After reaching the resuscitation time point, all sheep were subjected to another experimental protocol not concerning the hypothesis of the present study. According to that protocol, sheep were killed at the end of the experiments by intravenous injection of 4 mg·kg^− 1^ propofol and 200 ml potassium chloride solution (7.45%).

### Measurements

At baseline, at each stage of blood withdrawal, and after resuscitation, systemic haemodynamic variables were measured and blood samples were obtained for blood gas analysis.

Measured haemodynamic variables were cardiac index (CI), central venous pressure (CVP), heart rate (HR), mean arterial pressure (MAP), and stroke volume index (SVI). The CI was obtained by threefold bolus thermodilution using the PiCCO™ system and documenting the mean. MAP and the HR were read from the haemodynamic monitoring device. Blood gas analyses were performed with an ABL 725 Radiometer automatic blood gas analyser (Radiometer, Denmark).

### Optical coherence tomography angiography

**Fig. 1:**
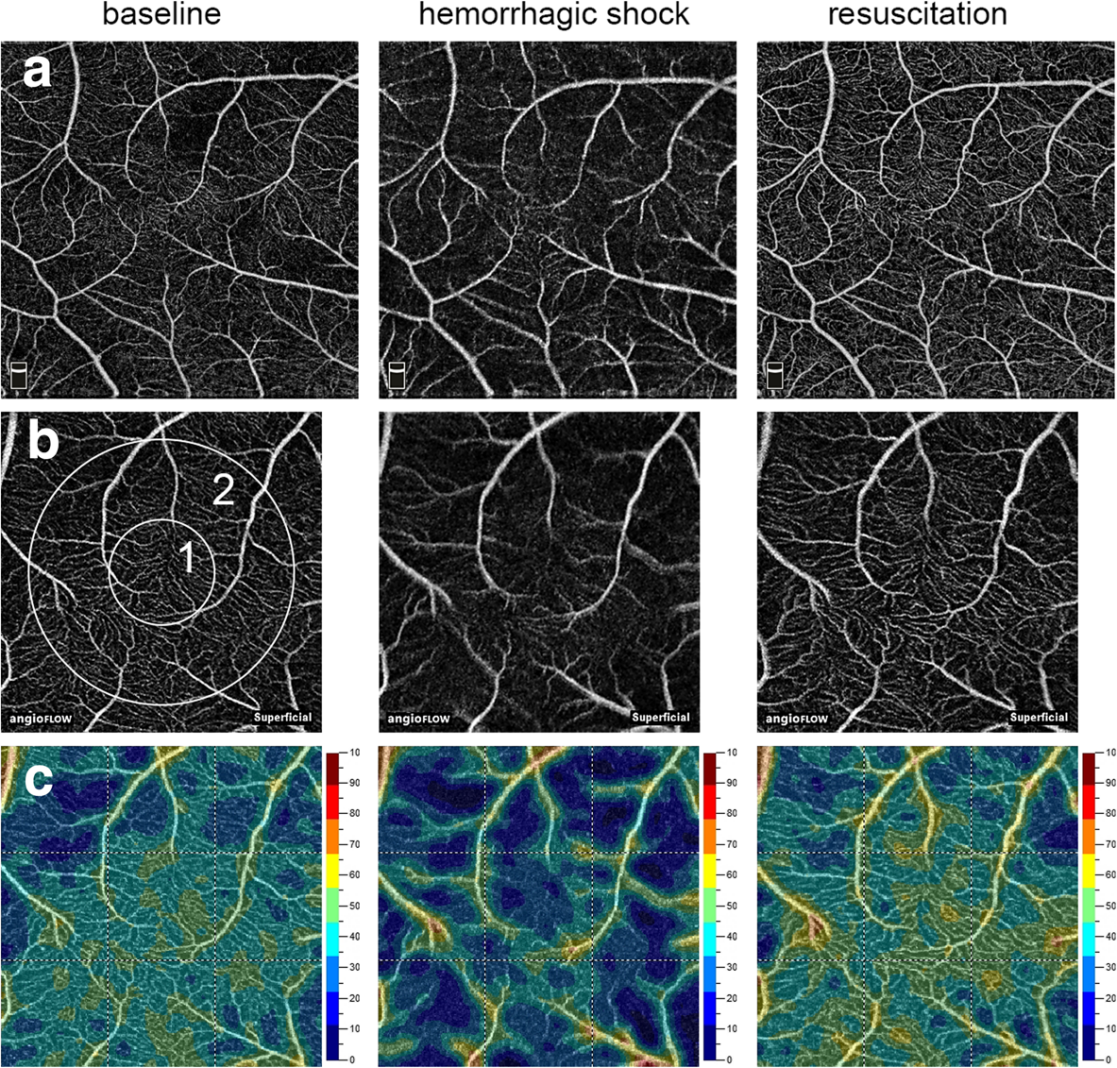
Optical coherence tomography angiograms of retina. Optical coherence tomography (OCT) angiograms (**a, b**) and colour-coded OCT angiograms (**c**) of same area of sheep retina at baseline, in haemorrhagic shock, and after resuscitation. Circle 1 indicates region used for calculation of flow density (central ring). Flow density (whole en face) is average flow density of circles 1 and 2. **a** 6 × 6 mm^2^ scans. **b, c** 3 × 3 mm^2^ scans

Imaging of the retinal vasculature was performed using the RTVue XR Avanti with AngioVue (Optovue Inc., Fremont, CA, USA). The instrument has an A-scan rate of 70,000 scans per second and uses a light source centred at 840 nm and a bandwidth of 50 nm. Each OCT angiography volume contained 304 × 304 A-scans. Split-spectrum amplitude-decorrelation angiography was used to extract the OCT-A information. The OCT-A technology has been described previously in detail in other studies [18, 20, 28, 29]. Briefly, OCT scans of a certain region of the retina were performed repeatedly and OCT images were then evaluated for changes. Static tissue shows little or no change, whereas blood flow in the retinal and choroidal vessels will result in differences between consecutive scans [29]. Before imaging, pupils were dilated with phenylephrine (Neosynephrin® 5%; Ursapharm Arzneimittel GmbH, Saarbrücken, Germany) and tropicamide 5 mg/ml (Mydriaticum® Stulln; Pharma Stulln GmbH, Stulln, Germany). The sheep were then placed in front of the OCT-A device. The cornea was lubricated before imaging and a lid speculum was used during imaging to keep the eye open. For orientation, a wide-field structural OCT scan and a 6 × 6 mm^2^ or 8 × 8 mm^2^ OCT-A scan were performed. To achieve high-quality imaging of the retinal microvasculature and for further analysis of the flow density, 3 × 3 mm^2^ scans of the same retinal area were performed during the entire experimental procedure (Additional file 1). The eye tracking system and the follow-up mode were activated during imaging to allow analysis of the same retinal area. Only OCT-A images of good quality and a signal strength index ≥ 55 were included. The software automatically segmented the retinal tissue into different retinal layers: superficial, deep, outer retina, and choriocapillaris. The segmentations of all examinations were checked before data analysis. The flow density data in the superficial OCT angiogram at baseline, after each step of bleeding, and after fluid resuscitation were then extracted and automatically analysed. In particular, the flow density of the central ring of the OCT angiogram (Flow density_Central_; circle 1 in Fig. 1) and the flow density whole en face (Flow density_WF_; the average flow density of circles 1 and 2 in Fig. 1) were calculated.

### Analysis of the conjunctival microcirculation

Conjunctival microcirculation was measured with an incident dark-field (IDF) video microscope (CytoCam™; Braedius Medical BV, Huizen, the Netherlands) at baseline, in haemorrhagic shock, and after resuscitation. For observation of the conjunctival microcirculation the opposite-side eye, which was not used for OCT angiography, was used to avoid affecting the conjunctival microcirculation with phenylephrine and tropicamide. At each time point at least five videos, each 5 s in length, were recorded. Videos of the conjunctival microcirculation were reviewed using previously described quality measures [30] and discarded if necessary. Analysis was conducted offline using dedicated software (AVA software version 3.2; Microvision Medical, Amsterdam, the Netherlands) according to the consensus conference criteria for analysis of the microcirculation in small vessels (< 20 μm) following established protocols [31–33]. At each time point, three to five high-quality videos of the conjunctival microcirculation were analysed in a blinded manner regarding the time point, and values of microvascular flow index (MFI), heterogeneity index (HI), total vessel density (TVD), perfused vessel density (PVD), and proportion of perfused vessels (PPV) were noted.

### Statistical analysis

Microsoft Excel 2010 was used for data arrangement. Statistical analyses were performed using IBM SPSS® Statistics 24 for Windows (IBM Corporation, Somers, NY, USA). Due to the small sample size, non-parametric tests were used. Data are presented as median with interquartile range. Comparisons between time points were made using Friedman’s test followed by a post hoc test (Dunn’s multiple comparison test). The degree of correlation between two variables was expressed as Spearman’s rank correlation coefficient. The global statistical significance level was set to 0.05. Inferential statistics are intended to be exploratory (i.e. as a basis for hypotheses), rather than confirmatory, and are interpreted accordingly.

## Results

**Fig. 2:**
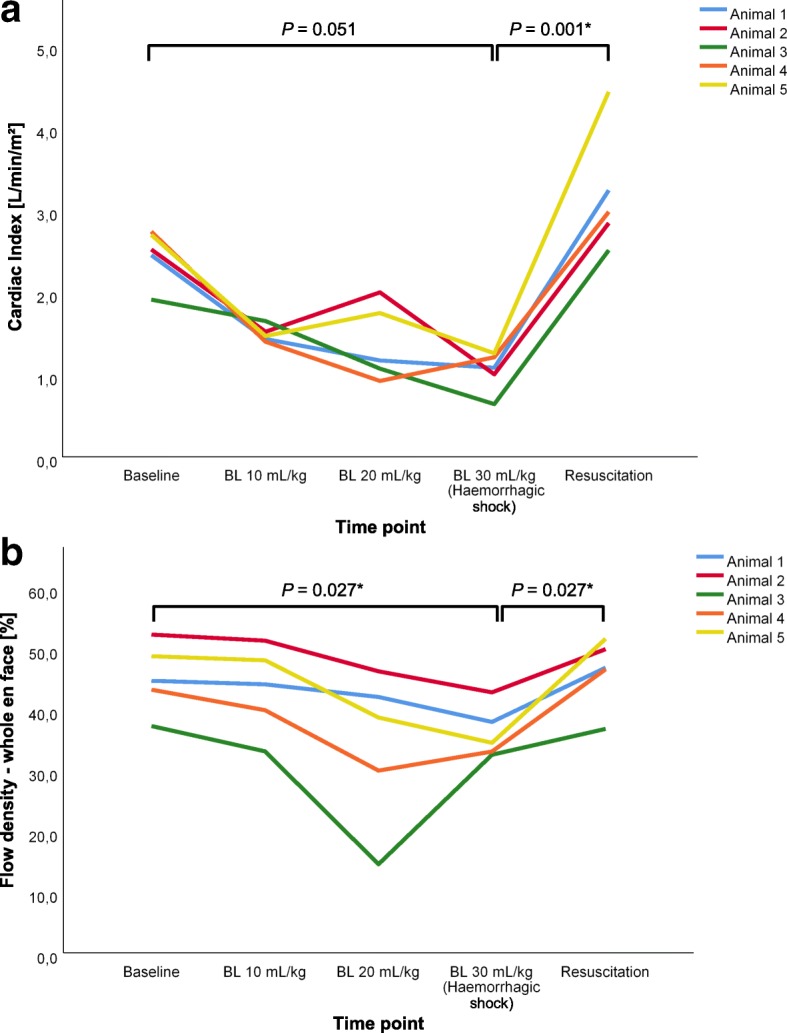
Cardiac index and retinal flow density during progressive haemorrhage and after resuscitation. Changes in **a** cardiac index and **b** retinal flow density (whole en face) during progressive haemorrhage and after resuscitation for each individual animal (*N* = 5). ^*^Significant difference. BL blood loss

At the end of the third step of blood withdrawal (haemorrhagic shock), a median 27 (24; 30) ml·kg^− 1^ blood was shed from the animals (animal 1, 27 ml·kg^− 1^; animal 2, 30 ml·kg^− 1^; animal 3, 30 ml·kg^− 1^; animal 4, 21 ml·kg^− 1^; animal 5, 27 ml·kg^− 1^). The MAP decreased significantly following blood loss (baseline 117 (108; 122) mmHg vs haemorrhagic shock 33 (30; 38) mmHg; *P* = 0.001) and increased almost to baseline levels after resuscitation (93 (76; 100) mmHg vs haemorrhagic shock; *P* = 0.164). The CI tended to decrease in haemorrhagic shock (1.1 (0.8; 1.2) L∙min^− 1^∙m^− 2^ vs baseline 2.5 (2.2; 2.7) L∙min^− 1^∙m^− 2^; *P* = 0.051) and increased significantly after resuscitation (3.0 (2.7; 3.8) L∙min^− 1^∙m^− 2^ vs haemorrhagic shock; *P* = 0.001; Fig. 2).

Further haemodynamic variables at baseline, during progressive haemorrhage, and after resuscitation are summarized in Table 1.

**Table 1:** Systemic haemodynamics and flow density of retina at baseline, during progressive haemorrhage, and after resuscitation

	Time point				
Parameter (unit)	Baseline	BL 10 ml∙kg^− 1^	BL 20 ml∙kg^− 1^	BL 30 ml∙kg^− 1^ (haemorrhagic shock)	Resuscitation
MAP (mmHg)	117 (108; 122)	80 (74; 98)	44 (37; 72)	33 (30; 38)*	93 (76; 100)
HR (1∙min^−1^)	86 (85; 94)	98 (84; 108)	107 (77; 118)	107 (92; 131)	128 (112; 139)*
CI (L∙min^−1^∙m^− 2^)	2.5 (2.2; 2.7)	1.5 (1.4; 1.6)	1.2 (1.0; 1.9)	1.1 (0.8; 1.2)	3.0 (2.7; 3.8)^#^
CVP (mmHg)	3 (1; 10)	6 (3; 9)	2 (0; 11)	0 (0; 6)	5 (1; 9)
SVI (ml∙m^−2^)	20 (22; 41)	17 (15; 23)	13 (9; 18)	11 (6; 21)	24 (22; 24)
Retinal microcirculation (OCT-A)					
Flow density_WF_ (%)	44.7 (40.3; 50.5)	44.1 (36.5; 49.7)	38.7 (22.2; 44.1)	34.5 (32.8; 40.4)*	46.9 (41.7; 50.7)^#^
Flow density_Central_ (%)	45.2 (36.7; 48.6)	43.4 (34.3; 46.4)	39.2 (21.3; 42.2)^§^	32.5 (32.2; 40.0)^§^	48.6 (38.4; 50.3)

Values presented as median (interquartile range)

*BL* blood loss, *CI* cardiac index, *CVP* central venous pressure, *Flow density*_*Central*_ flow density central ring, *Flow density*_*WF*_ flow density whole en face, *HR* heart rate, *MAP* mean arterial pressure, *OCT-A* optical coherence tomography angiography, *SVI* stroke volume index

^*^Significant difference vs baseline

^#^Significant difference vs haemorrhagic shock

^§^Significant difference vs after resuscitation

The central venous oxygen saturation decreased significantly at the time of haemorrhagic shock compared to baseline (baseline 86.5% (84.0; 88.5) vs haemorrhagic shock 38.8% (29.4; 46.1); *P* = 0.003) and increased to baseline levels after resuscitation (resuscitation 85.2% (79.7; 89.2) vs haemorrhagic shock; *P* = 0.027). Urine output and parameters of blood gas analyses at baseline, during progressive haemorrhage, and after resuscitation are summarized in Table 2.

**Table 2:** Urine output and parameters of blood gas analysis at baseline, during progressive haemorrhage, and after resuscitation

	Time point				
Parameter (unit)	Baseline	BL 10 ml∙kg^−1^	BL 20 ml∙kg^−1^	BL 30 ml∙kg^− 1^ (haemorrhagic shock)	Resuscitation
Urine output (ml∙h^−1^)	63 (85; 105)	20 (8; 30)	0 (0; 3)*	0 (0; 0)*	20 (20; 28)
Hb_a_ (g∙dl^−1^)	9.0 (8.9; 10.0)	9.2 (8.3; 9.6)^§^	8.5 (7.4; 8.6)	7.4 (7.1; 7.8)	5.7 (5.4; 6.2)*
Hct_a_ (%)	27.8 (27.5; 30.9)	28.6 (25.8; 29.6)^§^	26.3 (23.2; 27.0)	23.0 (22.2; 24.2)	18.1 (17.2; 19.4)*
pH	7.39 (7.35; 7.45)	7.44 (7.38; 7.47)	7.44 (7.39; 7.49)	7.41 (7.34; 7.46)	7.29 (7.26; 7.36)
Lactate (mmol∙l^−1^)	1.1 (0.6; 1.2)	0.7 (0.4; 0.9)^§^	1.0 (0.7; 1.3)	2.0 (1.7; 3.4)	3.8 (3.4; 4.5)
S_cv_O_2_	87 (84; 89)	68 (61; 76)	50 (33; 59)*	38.8 (29.4; 46.1)*	85.2 (79.7; 89.2)^#^

Values presented as median (interquartile range)

*BL* blood loss, *Hb*_*a*_ arterial haemoglobin concentration, *Hct*_*a*_ arterial haematocrit, *S*_*cv*_*O*_*2*_ central venous oxygen saturation

*Significant difference vs baseline

^#^Significant difference vs haemorrhagic shock

^§^Significant difference vs after resuscitation

With OCT-A imaging it was possible to visualize the optic disc and the retinal vasculature with very good image quality (Additional file 1). It was also possible to perform imaging of the same retinal area for several hours throughout the experimental procedure (Fig. 1). Figure 1 exemplifies the retinal vessels at baseline, during haemorrhagic shock, and after resuscitation. Flow density data of the same retinal area in a 3 × 3 mm^2^ scan were also evaluated throughout the experimental procedure. Flow density_WF_ in the superficial OCT angiogram of the retina decreased significantly after shock induction (baseline 44.7% (40.3; 50.5) vs haemorrhagic shock 34.5 (32.8; 40.4) %; *P* = 0.027) and recovered to baseline values after resuscitation (resuscitation 46.9% (41.7; 50.7) vs haemorrhagic shock; *P* = 0.027; Table 1 and Fig. 1).

The conjunctival microcirculation results are summarized in Table 3. The PPV decreased significantly at haemorrhagic shock compared to baseline values (baseline 100.0% (98.0; 100.0) vs haemorrhagic shock 72.0% (57.4; 76.3); *P* = 0.013) and increased substantially after resuscitation (resuscitation 98.7% (97.3; 99.1); *P* = 0.173 vs haemorrhagic shock). The MFI decreased in haemorrhagic shock (baseline 3.1 (3.0; 3.3) vs haemorrhagic shock 1.9 (1.9; 2.1); *P* = 0.034) and tended to increase after resuscitation (3.0 (3.0; 3.3) vs haemorrhagic shock; *P* = 0.081). Representative videos of the conjunctival microcirculation at baseline, in haemorrhagic shock, and after resuscitation are available as supplemental digital content (Additional files 2, 3 and 4).

**Table 3:** Parameters of conjunctival microcirculation at baseline, in haemorrhagic shock, and after resuscitation

	Time point		
Parameter (unit)	Baseline	Haemorrhagic shock	Resuscitation
TVD (mm∙mm^−2^)	15.4 (13.7; 17.3)	18.5 (15.1; 19.5)	16.3 (15.3; 18.6)
PVD (mm∙mm^−2^)	15.4 (13.4; 17.3)	12.1 (10.0; 13.8)	16.0 (15.2; 18.3)
PPV (%)	100.0 (98.0; 100.0)	72.0 (57.4; 76.3)*	98.7 (97.3; 99.1)
MFI	3.1 (3.0; 3.3)	1.9 (1.9; 2.1)*	3.0 (3.0; 3.3)
HI	0.10 (0.05; 0.16)	0.51 (0.24; 0.73)*	0.12 (0.33; 0.37)

Values presented as median (interquartile range)

*HI* heterogeneity index, *MFI* microvascular flow index, *PPV* proportion of perfused vessel, *PVD* perfused vessel density, *TVD* total vessel density

*Significant difference vs baseline

**Fig. 3:**
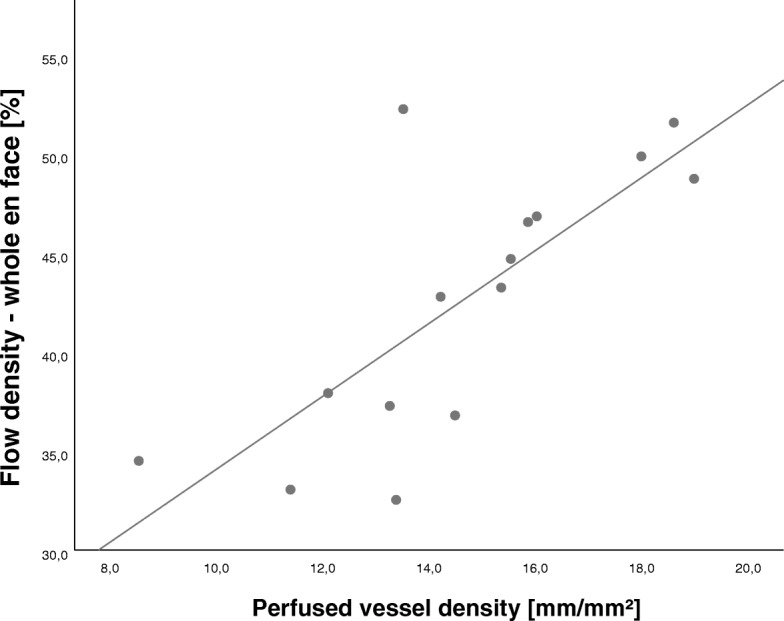
Scatter diagram of retinal flow density (whole en face) and conjunctival perfused vessel density. Dots represent measurements at baseline, haemorrhagic shock, and resuscitation in the five animals

Flow density_WF_ in the superficial retinal OCT angiogram correlated significantly with parameters of the conjunctival microcirculation (PVD: Spearman’s rank correlation coefficient ρ = 0.750, *P* = 0.001; PPV: Spearman’s rank correlation coefficient ρ = 0.620, *P* = 0.014; MFI: Spearman’s rank correlation coefficient ρ = 0.679, *P* = 0.005; see Fig. 3) and haemodynamic parameters (CI: Spearman’s rank correlation coefficient ρ = 0.693, *P* < 0.001; MAP: Spearman’s rank correlation coefficient ρ = 0.594, *P* = 0.002; SVI: Spearman’s rank correlation coefficient ρ = 0.639, *P* = 0.002).

## Discussion

The present study shows for the first time that OCT-A has potential as a novel technology for non-invasive and contactless monitoring of the retinal microcirculation during progressive haemorrhagic shock and resuscitation. Flow density_WF_ measured by OCT-A decreased significantly in haemorrhagic shock and recovered after resuscitation. The changes in Flow density_WF_ measured with OCT-A correlated with parameters of systemic haemodynamics and conjunctival microcirculation. The latter was analysed using IDF video microscopy and is the current gold standard for microcirculatory analyses.

In the current study, induction of haemorrhagic shock by removal of a large amount of blood resulted in typical changes in systemic haemodynamics, with reductions in cardiac index and a decrease in perfusion pressure. The drop in central venous oxygen saturation and increasing lactate concentrations may be interpreted as signs of tissue hypoperfusion and cellular oxygen deficit. To compensate, sheep reacted with an increase in heart rate and by recruiting fluid from the interstitium, as indicated by haemodilution (reflected by decreases in haematocrit and haemoglobin levels). These changes were accompanied by a reduced microvascular flow (MFI) and a drop in capillary perfusion (PPV) of the conjunctival microcirculation. These observations confirmed those obtained in previous studies on sheep in haemorrhagic shock [25, 26]. We now show that these changes seen in systemic and microcirculatory variables during haemorrhagic shock and after resuscitation are closely correlated to OCT-A data of the retina. As the ophthalmic artery arises from the internal carotid artery, altered retinal perfusion may indicate impaired cerebral microcirculation. Many previous studies have shown a close relationship between cerebral events and qualitative measurements of retinal microvasculature [11–13, 15]. In this context, it should be mentioned that the brain and retina are controlled by a local autoregulatory mechanism in which the blood flow is regulated according to metabolic needs and oxygen consumption demands despite moderate variations in perfusion pressure [34]. In the current study, the relative maintenance of the retinal Flow density_WF_ after blood loss of 10 ml∙kg^− 1^ body weight in all sheep (Fig. 2), while MAP was reduced by approximately one third, may be interpreted as a MAP within the retinal autoregulatory range, while further blood loss undercut the retinal autoregulatory threshold, which was reflected by a drop in Flow density_WF_ in the current study. In this context, Park et al. [35] presented preliminary results of OCT-A analyses during haemorrhagic shock in rats and reported a constant retinal blood flow despite a drop in MAP. Although exact values were not reported, it may be hypothesized that the MAP remained above the threshold of autoregulation since they removed 40% of rat blood volume [35], as compared to about 60% in the present study. Against this background, Ono et al. [36] monitored the cerebral autoregulation response during cardiac surgery using near-infrared spectroscopy and showed that blood pressure excursions below the cerebral autoregulation threshold were associated with acute kidney injury. Therefore, further research may investigate the association between retina perfusion, assessed by OCT-A, and organ (dys)function.

Future clinical use of OCT-A to quantify tissue perfusion could be valuable in situations in which classical surrogates for monitoring of tissue perfusion, such as haemodynamic parameters or urine output, do not always adequately reflect the level of tissue perfusion (e.g. prolonged haemorrhagic or septic shock). Optimizing macro-haemodynamic parameters in this setting does not necessarily ensure adequate tissue perfusion [5–9]. However, the utility of OCT-A for these important issues needs to be evaluated in further experimental and clinical studies.

The present feasibility study demonstrates for the first time that, as a novel, non-invasive, and contactless technology, OCT-A can monitor microcirculatory perfusion during shock states in large animals. It may also have the potential to overcome some critical issues in monitoring microvascular perfusion, which impede the clinical use of methods to monitor the microcirculation [37]. In this context, a quantitative evaluation of the retinal microcirculation can be automatically performed with OCT-A without the need for intravenous dye injection [10]. The reproducibility of flow density measurements using OCT-A was evaluated in healthy subjects, in patients with different ocular and systemic diseases, and in different animal models [20, 21, 24, 28, 38]. In addition, by taking simple precautions, such as using a lid speculum, imaging in mydriasis, and lubrication of the cornea, blood flow can be analysed in the same retinal vessels of the same retinal area over the entire experimental period, which is not possible with most other methods of microvascular monitoring.

The fact that retinal perfusion, conjunctival microcirculation, and systemic haemodynamics showed a very close correlation is probably owed to the relatively homogeneous impairment of tissue perfusion in the early phase of haemorrhagic shock. It must be expected that results would be more heterogeneous in septic or other types of distributive shock. Heterogeneous changes in the microcirculation have been described previously [39, 40].

The utility of the innovative technology presented here in an intensive care setting remains to be evaluated in further experimental and clinical studies. In particular, the OCT-A instrument used in the current study needs a horizontal orientation of the ocular bulb, which is not feasible in critically ill humans. In this regard, Heidelberg Engineering, for example, recently presented the OCT FLEX device (with OCT-A module), which enables imaging on recumbent patients. However, there are further problems to be solved before OCT-A can be used routinely in critically ill patients as, for example, imaging in miosis (stimulant monitoring of the pupil reaction) or imaging through different media opacities (especially cataract in elderly patients).

### Limitations of the current study

The main limitation of the present study is the small number of animals which were included in the study. This was due to a time-limited availability of the used OCT device. The results should show the feasibility of OCT-A to detect changes in retinal blood flow in haemorrhagic shock and were hypothesis generating in nature. Second, HES solutions were used for fluid resuscitation, which are contraindicated at present in critically ill patients due to suspected increases in renal failure [41], but may be used as plasma volume replacement following acute (sudden) blood loss (as was present in this study). In the current study, HES was chosen to investigate the immediate effect of fluid resuscitation on retinal perfusion and to show the feasibility of OCT-A to detect these changes. In this regard, the resuscitation protocol differs from current recommendations for management of major bleeding and haemorrhagic shock [42]. Another limitation of the study is the measurement of the conjunctival microcirculation instead of the sublingual microcirculation, which is the common region for microcirculatory measurements in critically ill patients [1, 6, 32]. Due to the head attachment of the sheep in prone position to the OCT device, it was not possible to measure the sublingual microcirculation in parallel with retinal perfusion. However, changes in sheep conjunctival microcirculation in haemorrhagic shock were shown to be comparable to changes in the sublingual microcirculation [43].

## Conclusions

The current study shows for the first time that OCT-A is feasible to monitor retinal perfusion in sheep during experimental haemorrhagic shock and fluid resuscitation. We show that changes in retinal perfusion examined using OCT-A correlate with changes in systemic haemodynamic parameters and with variables of conjunctival microcirculation. This makes OCT-A a promising tool for in-vivo monitoring of the central microcirculation in critical illness. Further studies are needed to evaluate the utility of this technology in clinical practice.

## Additional files

Additional file 1: Optical coherence tomography of retina. **A.** 4.5 × 4.5 mm^2^ scan of optic nerve head (ONH) showing large retinal vessels arising from ONH. **B.** 6 × 6 mm^2^ scan of retina showing retinal vessels; scans used for orientation alone. **C.** 3 × 3 mm^2^ scan of retina providing high-quality visualization of the retinal microcirculation. (TIF 573 kb)

Additional file 2: Video of conjunctival microcirculation at baseline. Conjunctival microcirculation at baseline measured with incident dark-field (IDF) video microscope. (MP4 3467 kb)

Additional file 3: Video of conjunctival microcirculation in haemorrhagic shock. Conjunctival microcirculation in haemorrhagic shock measured with incident dark-field (IDF) video microscope. (MP4 4106 kb)

Additional file 4: Video of conjunctival microcirculation after resuscitation. Conjunctival microcirculation after resuscitation with hydroxyethyl starch solution (HES 130/0.4) measured with incident dark-field (IDF) video microscope. (MP4 3499 kb)

## Abbreviations

CI: Cardiac index
CVP: Central venous pressure
Flow density_Central_: Flow density central ring
Flow density_WF_: Flow density whole en face
HES: Hydroxyethyl starch solution
HI: Heterogeneity index
HR: Heart rate
IDF: Incident dark field
MAP: Mean arterial pressure
MFI: Microvascular flow index
OCT-A: Optical coherence tomography angiography
PPV: Proportion of perfused vessel
PVD: Perfused vessel density
SVI: Stroke volume index
TVD: Total vessel density
