# Supplementary figures and images for: Feasibility of optical coherence tomography angiography to assess changes in retinal microcirculation in ovine haemorrhagic shock
Source: Crit Care. 2018 May 29;22:138. doi: 10.1186/s13054-018-2056-3 (PMC5975442; doi:10.1186/s13054-018-2056-3)

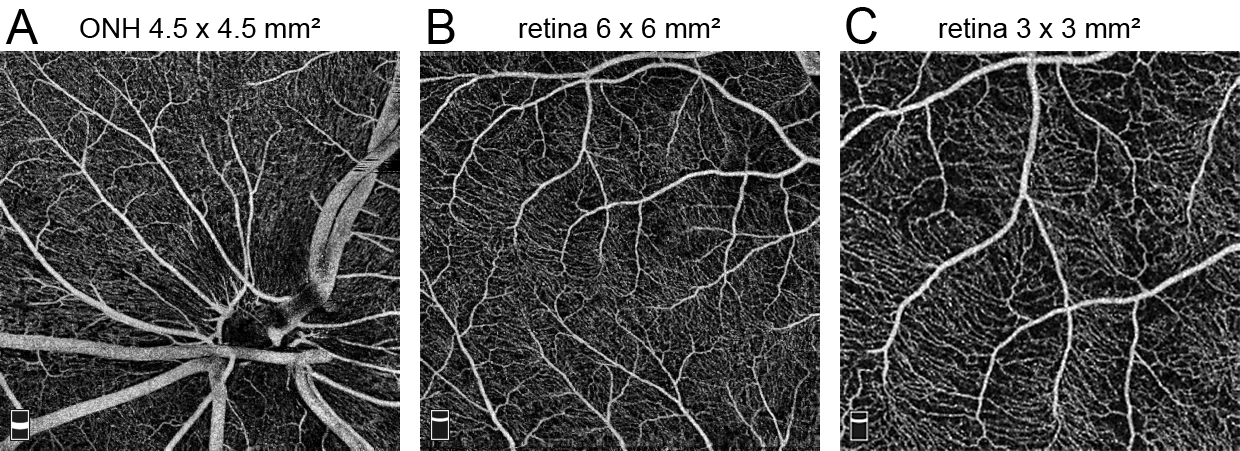

Supplement: Supplementary file 1 — Optical coherence tomography of retina. A. 4.5 × 4.5 mm2 scan of optic nerve head (ONH) showing large retinal vessels arising from ONH. B. 6 × 6 mm2 scan of retina showing retinal vessels; scans used for orientation alone. C. 3 × 3 mm2 scan of retina providing high-quality visualization of the retinal microcirculation. (TIF 573 kb) [file 13054_2018_2056_MOESM1_ESM.tif]
